# Oral lichen planus treated with upadacitinib: A case series

**DOI:** 10.1016/j.jdcr.2025.05.041

**Published:** 2025-07-01

**Authors:** Christiaan H. Noot, Annika M. Hansen, Zachary Frost, Jamie L.W. Rhoads, Christopher M. Hull, John J. Zone, Zachary H. Hopkins

**Affiliations:** a Department of Dermatology, Autoimmune Skin Diseases Clinic, University of Utah Spencer F. Eccles School of Medicine, Salt Lake City, Utah; b Noorda College of Osteopathic Medicine, Provo, Utah

**Keywords:** dermatology, JAK inhibitor, lichen planus, LP, OLP, oral lichen planus, upadacitinib

Oral lichen planus (OLP) is a chronic inflammatory disease affecting the oral mucosa. It impacts approximately 0.5% to 2% of the global population and can increase the risk of oral squamous cell carcinoma.^1^^,^^2^ It clinically manifests as reticular, erosive, bullous, or plaque-like lesions, primarily affecting the buccal mucosa, tongue, and gingiva.^1^ Although first-line treatment is topical therapy (corticosteroids or calcineurin inhibitors), moderate to severe cases often require systemic therapies to achieve disease control. Since OLP is driven by an interferon gamma-dominated response mediated via the Janus kinase/signal transducer and activator of transcription pathway, Janus kinase inhibitors may be a promising modality for recalcitrant OLP. However, little data exist regarding the effect of Janus kinase inhibitors on OLP.^3^

This case series included patients with biopsy-proven OLP seen at the University of Utah between January 1, 2023, and January 1, 2025, with severe OLP who received upadacitinib. Data collected included demographic information, clinical characteristics, treatment details, and outcomes (Table I). All information was obtained through a retrospective chart review. Treatment outcomes were classified as clearance (no erosions, no erythema, no pain), large improvement (no erosions, improved erythema, improved pain), small improvement (improved erosions, improved erythema, improved pain), and no response. Institutional review board approval for the case series and the retrospective analyses was obtained from the University of Utah School of Medicine (#IRB_00076927). All statistics are descriptive and were performed in Stata V18.0 (StataCorp).

**Table I:** Patient characteristics and treatment

Patient	Age (y)	Disease duration before upadacitinib (y)	Involved oral sites	Prior systemic treatments	Concomitant medications	Treatment outcome	Treatment duration with upadacitinib (d)
1	51	2	Mucosal lip, upper gingiva	MTX, HCQ, MP	Topicals, HCQ	Clearance	170
2	52	3	Tongue, gingiva, buccal mucosa	MTX, HCQ, IVIG, colchicine	Topicals, IVIG∗	Clearance	232
3	85	4	Buccal mucosa, tongue	Cyclosporine, apremilast	Topicals	Clearance	394
4	56	1.5	Gingiva, hard palate	None	None	Clearance	344
5	73	12	Mucosal lip, tongue, buccal mucosa	MTX, HCQ, AZA, MP, cyclosporine	Topicals, HCQ	Large improvement	610
6	72	4	Mucosal lip, tongue	HCQ, MP, acitretin	Topicals	Large improvement	183
7	73	11	Gingiva, hard palate	Tacrolimus, HCQ, tildrakizumab, AZA, MP, cyclosporine, apremilast	Topicals, azathioprine	Large improvement	267
8	66	29	Buccal mucosa, hard palate, tongue	Tacrolimus, MTX, HCQ, thalidomide, adalimumab, cyclosporine, apremilast, tofacitinib	Tildrakizumab, prednisone	Clearance	281
9	75	12	Vermillion, lateral tongue	MTX, HCQ, tildrakizumab, AZA, MP, cyclosporine, apremilast	Dexamethasone, prednisone	Small improvement	569
10	74	3	Buccal mucosa, gingiva, tongue	MTX, HCQ, cyclosporine, apremilast	None	Small improvement	155

*AZA*, Azathioprine; *HCQ*, hydroxychloroquine; *IVIG*, intravenous immunoglobulin; *MP*, mycophenolate; *MTX*, methotrexate.

∗ Patient was on IVIG to treat a separate diagnosis.

We identified 10 patients meeting the criteria. All patients were female, and the mean age was 68.2 years. All patients had symptomatic inflammatory disease and 9/10 patients had erosive disease. 7/10 patients had only oral disease, whereas patient 8 had vulvar lichen planus, and patient 4 was being treated primarily for severe vulvar lichen sclerosis. All patients failed topical therapy. Failed systemic medications included hydroxychloroquine, colchicine, methotrexate, thalidomide, tildrakizumab, adalimumab, azathioprine, mycophenolate, cyclosporine, apremilast, tacrolimus, and prednisone. Of note, patient 8 previously trialed tofacitinib (10 mg twice a day) with inadequate disease control. The mean disease duration (years) and number of failed systemic medications before starting upadacitinib were 8.2 and 4.3, respectively. After treatment with either 15 mg or 30 mg of upadacitinib daily, all patients demonstrated either clearance (*n* = 5, 50%), large improvement (*n* = 3, 30%), or small improvement (*n* = 2, 20%). Patients 2 and 3 exhibited symptoms concerning for burning mouth syndrome (complete OLP clinical clearance, with residual pain) after treatment. Patients 4 and 8 had vulvar disease clear alongside oral disease. Patients 8 and 9 had successful treatment with upadacitinib in combination with prednisone, though both patients were able to maintain improvement on upadacitinib alone. The mean response time to treatment and follow-up was 45.6 and 320 days, respectively. The mean number of concomitant treatments, excluding topical therapy, was 0.9. These included hydroxychloroquine, intravenous immunoglobulin, tildrakizumab, and systemic corticosteroids. There were no reported adverse events or treatment discontinuation due to side effects.

This study provides early data suggesting that upadacitinib may be a promising option for recalcitrant OLP. These results are consistent with case reports of upadacitinib successfully treating OLP.^4^^,^^5^ Limitations include small sample size, demographic homogeneity, and lack of a control group.

## Conflicts of interest

None disclosed.
